# Antibody persistence and safety after heterologous boosting with orally aerosolised Ad5-nCoV in individuals primed with two-dose CoronaVac previously: 12-month analyses of a randomized controlled trial

**DOI:** 10.1080/22221751.2022.2155251

**Published:** 2022-12-15

**Authors:** Lairun Jin, Rong Tang, Shipo Wu, Xiling Guo, Haitao Huang, Lihua Hou, Xiaoqin Chen, Tao Zhu, Jinbo Gou, Jin Zhong, Hongxing Pan, Lunbiao Cui, Yin Chen, Xin Xia, Jialu Feng, Xue Wang, Qi Zhao, XiaoYu Xu, Zhuopei Li, Xiaoyin Zhang, Wei Chen, Jingxin Li, Fengcai Zhu

**Affiliations:** a School of Public Health, Southeast University, Nanjing, People’s Republic of China; b National Health Commission (NHC) Key Laboratory of Enteric Pathogenic Microbiology (Jiangsu Provincial Center for Disease Control and Prevention), Nanjing, People’s Republic of China; c Beijing Institute of Biotechnology, Academy of Military Medical Sciences, Beijing, People’s Republic of China; d Cansino Biologics Inc., Tianjin, People’s Republic of China; e Donghai County Center for Disease Control and Prevention, Lianyungang, Jiangsu, People’s Republic of China; f School of Public Health, Nanjing Medical University, Nanjing, People’s Republic of China; g Vazyme Biotech Co., Ltd, Nanjing, People’s Republic of China; h Institute of Global Public Health and Emergency Pharmacy, China Pharmaceutical University, Nanjing, People’s Republic of China

**Keywords:** COVID-19 vaccines, booster dose, long-term immunity, heterologous, orally administration

## Abstract

Antibody persistence and safety up to 12 months of heterologous orally administered adenovirus type-5 vector-based COVID-19 vaccine (Ad5-nCoV) in individuals who were primed with two-dose inactivated SARS-CoV-2 vaccine (CoronaVac) previously, has not been reported yet. This randomized, open-label, single-centre trial included Chinese adults who have received two-dose CoronaVac randomized to low-dose or high-dose aerosolised Ad5-nCoV group, or CoronaVac group. In this report, we mainly evaluated the geometric mean titres (GMTs) of neutralizing antibodies (NAbs) against live wild-type SARS-CoV-2 virus and omicron BA.4/5 pseudovirus at 12 months after the booster dose and the incidence of serious adverse events (SAEs) till month 12. Of 419 participants, all were included in the safety analysis and 120 (28.64%) were included in the immunogenicity analysis. Serum NAb GMT against live wild-type SARS-CoV-2 was 204.36 (95% CI 152.91, 273.14) in the low-dose group and 171.38 (95% CI 121.27, 242.19) in the high-dose group at month 12, significantly higher than the GMT in the CoronaVac group (8.00 [95% CI 4.22, 15.17], *p* < 0.0001). Serum NAb GMT against omicron BA.4/5 pseudovirus was 40.97 (95% CI 30.15, 55.67) in the low-dose group and 35.08 (95% CI 26.31, 46.77) in the high-dose group at month 12, whereas the GMT in the CoronaVac group was below the lower limit of detection. No vaccine-related SAEs were observed. Orally administered aerosolised Ad5-nCoV following two-dose CoronaVac priming has a good safety profile and is persistently more immunogenic than three-dose CoronaVac within 12 months after the booster dose.

Trial registration: ClinicalTrials.gov identifier: NCT05043259..

## Introduction

The Coronavirus Disease 2019 (COVID-19) pandemic caused by severe acute respiratory syndrome coronavirus 2 (SARS-CoV-2) is profoundly affecting life around the world. Globally, as of 25 October 2022, there have been more than 0.6 billion confirmed cases of COVID-19, including nearly 6.5 million deaths, and the number of cases continues to rise. Safe and effective vaccination strategies are urgently needed to control the COVID-19 pandemic [1]. As of 19 October 2022, 11 vaccines have been granted emergency use by WHO, all of which were administered intramuscularly [2].

The emergence and rapid spread of SARS-CoV-2 variants of concern and waning immunity over time are challenging the effectiveness of vaccines [3,4]. In view of this emerging evidence, the WHO recommends that homologous (same vaccine platform) or heterologous (different vaccine platform) booster (third) doses should be offered 4–6 months after completion of the primary vaccination series, especially for inactivated vaccines [5]. Previous studies supported a heterologous booster regimen following the completion of two-dose CoronaVac (the Sinovac inactivated whole-virion SARS-CoV-2 vaccine) [6–10]. However, these reports were limited to 28 days or 3 months after the booster dose. The long-term immunity of heterologous prime-boost regimens is currently unknown.

In our previously reported study [11], a heterologous booster vaccine with an orally administered aerosolised Ad5-nCoV (by CanSino Biologics, China) induced substantial serum neutralizing antibody (NAb) titres against SARS-CoV-2, which were 6.7-fold and 10.7-fold stronger than a homologous CoronaVac booster at day 14 and day 28 after the booster dose, respectively. Participants who inhaled 0.1 and 0.2 mL of aerosolized Ad5-nCoV had NAb GMT peaks of 6054.1 and 4221.3 IU/mL against live SARS-CoV-2 virus, respectively, at day 28 after the booster vaccination, which was higher than three doses of BNT162b2 (955.7 IU/mL) using the WHO international standard. In addition, a heterologous booster vaccine with an orally administered aerosolised Ad5-nCoV was safe and had lower adverse reactions than a homologous boost with CoronaVac in adults who have previously received two-dose CoronaVac. However, antibody persistence and long-term safety of this vaccination regimen are not been reported yet. Here, we updated the serological and safety data to 12 months following the boost dose in this trial.

## Methods

### Study design and samples

We did a randomized, open-label, single-centre trial on the safety and immunogenicity of heterologous boost immunization with an orally administered aerosolised Ad5-nCoV after two-dose priming with an inactivated SARS-CoV-2 vaccine in Chinese adults aged 18 years and older (ClinicalTrials.gov number: NCT05043259), which has been reported previously [11]. Briefly, 419 healthy participants without the history of SARS-CoV-2 infection who had received two-dose CoronaVac as primary immunization were randomly assigned (1:1:1) to one of three groups: 140 participants received a heterologous booster immunization with aerosolised Ad5-nCoV at 0.1 mL in the low-dose group, 139 received a heterologous booster immunization with aerosolised Ad5-nCoV at 0.2 mL in the high-dose group, and 140 received a homologous intramuscular booster immunization at 0.5 mL in the CoronaVac group. The trial protocol was approved by the Research Ethics Committee of Jiangsu Provincial Center for Disease Control and Prevention (JSVCT127) and was conducted following the principles of the Declaration of Helsinki, ICH Good Clinical Practice guidelines and local guidelines. Written informed consent was obtained from all participants. We have previously reported the safety and immunogenicity within 28 days post-boost [11]. In this report, the incidence of serious adverse events (SAEs) till the 12 months after the booster dose was considered as the safety endpoint. While the NAb GMTs against live wild-type SARS-CoV-2 virus and wild-type SARS-CoV-2 RBD-specific IgG antibody responses at months 3, 6 and 12 after the booster dose were evaluated as immunogenicity endpoints. Moreover, we revealed the NAb responses against live omicron BA.1 subvariant at months 3 and 6, and NAb responses against omicron BA.4/5 pseudovirus at months 3, 6 and 12 in each of the three treatment groups as post-hoc analyses. We also reported the pregnancy events that occurred 12 months after booster vaccination.

### Serologic assays

Serum samples were collected from 120 participants (first 40 participants in each group [11]) in the sub-cohort for antibody persistency analysis. NAb titres against live wild-type SARS-CoV-2 and omicron BA.1 subvariant were determined by using a cytopathic effect-based microneutralization assay with a wild­type SARS-CoV-2 virus isolate BetaCoV/Jiangsu/JS02/2020 (GISAID EPI_ISL_411952) and an omicron (B.1.1.529) variant isolate hCoV-19/Jiangsu/JS01/2022 (GISAID EPI_ISL_12511653) (BA.1) in Vero­E6 cells (National Collection of Authenticated Cell Cultures, National Academy of Science, China). The serum dilution for microneutralization assay was 1:8–1:8192 and then mixed with the equal volume of virus solution to reach a 50% tissue culture infectious dose of 100 per well. The reported titres were the reciprocal of the highest sample dilution observed by an inverted microscope protecting at least 50% of the cells from cytopathy. The seropositivity for NAbs was defined as titre ≥1:8. We used the WHO International Standard (NIBSC code 20/136) as the reference for the cytopathic effect-based microneutralization assays. We also measured the NAb titres against omicron BA.4/5 subvariant induced by vaccination using pseudovirus neutralization tests (an HIV pseudovirus system expressing the spike glycoprotein) with a detectable NAb titre ≥1:30 [12]. We used the commercial Anti-SARS-CoV-2 RBD-IgG ELISA kit (Vazyme Medical Technology, Nanjing, China) to measure wild-type SARS-CoV-2 RBD-specific IgG responses (RU/mL) with a cutoff titre of 1:10 [11].

### Statistical analysis

The sample size calculation was based on the hypothesis that a booster vaccination with aerosolised Ad5-nCoV following two doses of CoronaVac could elicit a non-inferior or superior serum NAbs response at 14 days after the booster dose compared with a third dose of CoronaVac does, which has been reported previously [11]. Serum NAbs titres against the wild-type SARS-CoV-2 virus and omicron (B.1.1.529) variant at day 28, month 3, month 6 and month 12 were measured in a subgroup of the first 40 participants out of 140 participants in each group. The serum NAbs and RBD-specific IgG antibodies GMTs were calculated with the two-sided 95% confidence intervals (CIs) on the basis of the t-distribution of the log-transformed titres and were then back-transformed to the original scale. Geometric mean fold increases (GMFIs) of NAbs and RBD-specific binding antibodies and GMT ratios of the heterologous boost group versus the homologous boost group were also calculated. Data below the detection limit were assigned a value half of that limit and data above the highest detection limit were assigned a value of the threshold. *χ*^2^ test and Fisher’s exact test were used to analyse categorical data when appropriate. Analysis of variance was used to analyse the log-transformed antibody titres and the Wilcoxon rank-sum test was used for non-normal distributed data. SAS (version 9.4) and GraphPad Prism (version 8.0.1) were used for statistical analyses.

## Results

### Trial profile and demographic characteristics of the participants

**Figure 1.**
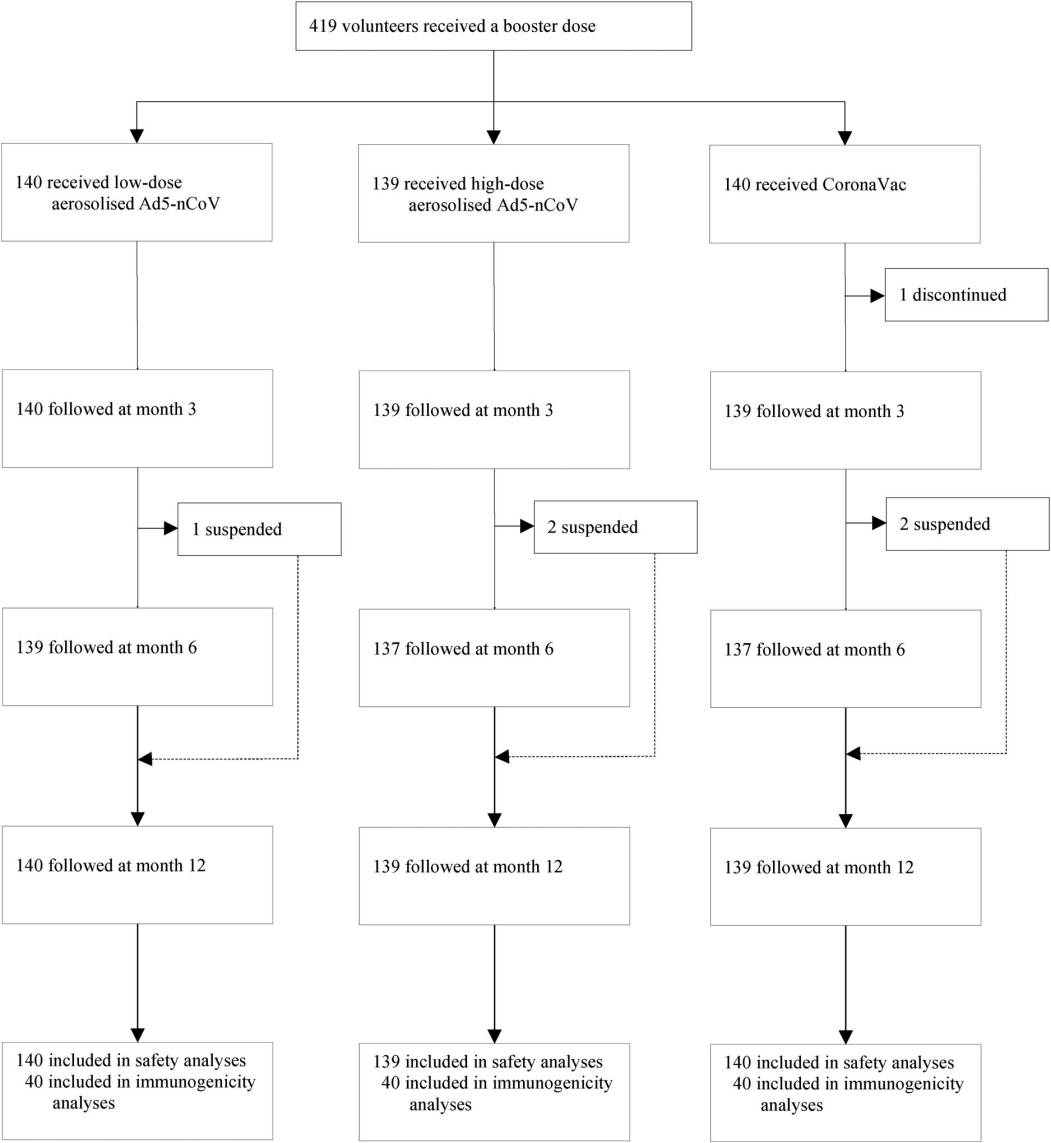
Study profile. Ad5-nCoV = orally administered aerosolised adenovirus type-5 vector-based COVID-19 vaccine carrying full-length SARS-CoV-2 spike gene. CoronaVac = intramuscularly administered inactivated whole-virion SARS-CoV-2 vaccine.

Of the 419 participants, 418 (99.76%) completed the 12-month follow-up, and all (100.00%) were involved in the safety analysis cohort. In the sub-cohort of antibody persistence analysis, 120 (100.00%) participants donated blood samples on day 28 after the booster dose, and 118 (98.33%), 114 (95.00%), and 91 (75.83%) participants contributed blood samples at months 3, 6, and 12, respectively (Figure 1). In both cohorts, the median time interval between the second prime dose of CoronaVac and the booster was 5.00 (IQR 5.00, 5.00) months. Demographic characteristics of the participants were comparable across the groups in both cohorts (Table S1).

### NAbs against live wild-type SARS-CoV-2

**Figure 2.**
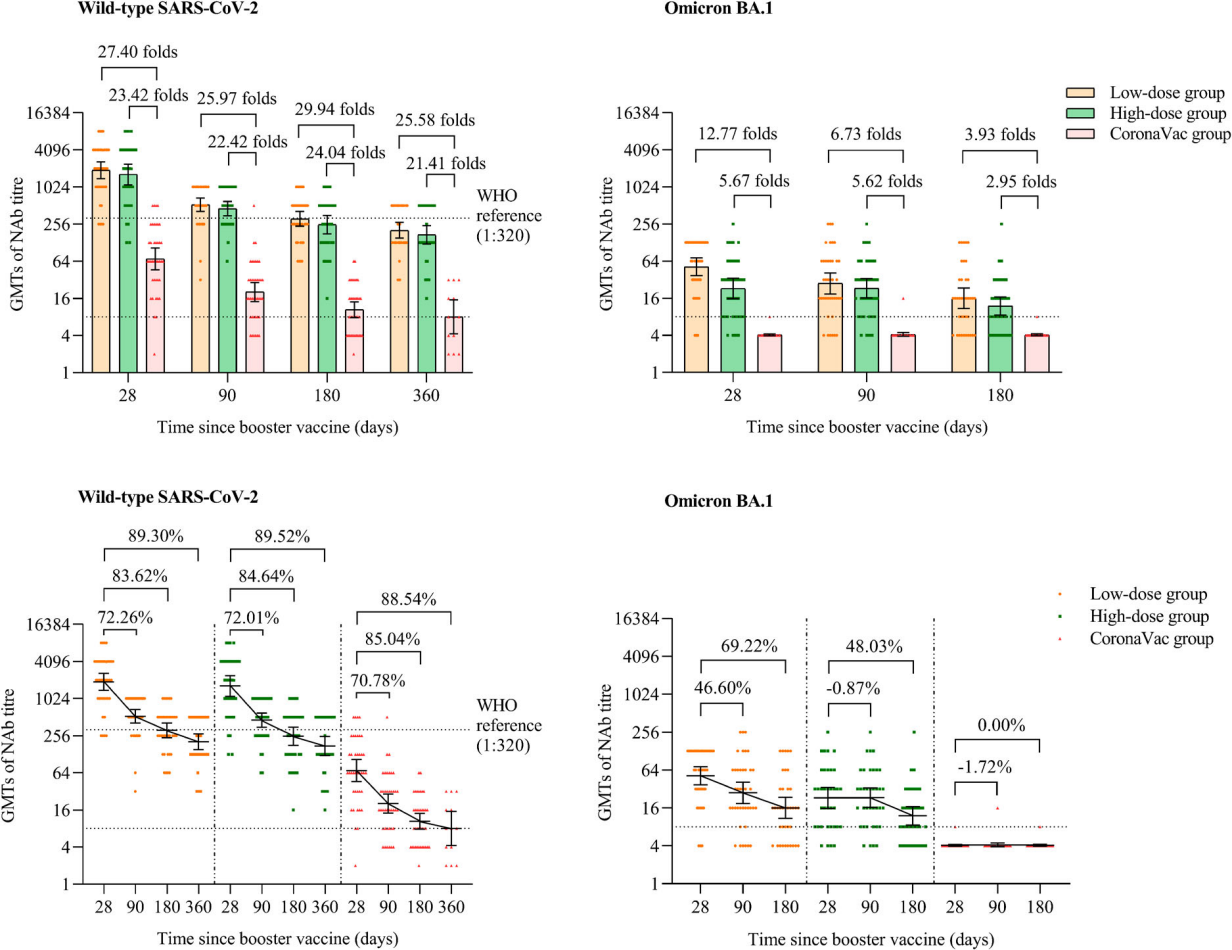
NAbs against live wild-type SARS-CoV-2 and omicron BA.1 subvariant after a booster vaccination. Horizontal bars show GMTs and error bars show 95% confidence intervals. Data (folds) above the bars are the NAb GMT ratios of the homologous boost group to the heterologous boost group. Long lines connecting the GMTs of adjacent groups indicate trends in NAbs over days after a booster vaccination. Data (%) above the bars show the percent reduction in GMTs of NAb titres at day 90, day 180 and day 360 post-boost compared to day 28. All horizontal dotted lines denote the cutoff levels for positivity (1:8) or the levels of WHO international standard (NIBSC code 20/136). Data below the cutoff level were assigned half the limit. GMT = geometric mean titre in serum. NAb = neutralizing antibody.

Participants in the low-dose and high-dose aerosolised Ad5-nCoV groups showed NAb GMTs against live wild-type SARS-CoV-2 of 1910.54 (95% CI 1391.98, 2622.28), 1635.03 (95% CI 1112.16, 2403.73) at day 28, 530.06 (95% CI 412.49, 681.12), 457.57 (95% CI 349.40, 599.22) at month 3, 312.88 (95% CI 237.71, 411.82), 251.12 (95% CI 178.16, 353.96) at month 6, and 204.36 (95% CI 152.91, 273.14), 171.38 (95% CI 121.27, 242.19) at month 12, which were significantly higher than those of the CoronaVac group, with GMT ratios of 25.97 and 22.42 folds at month 3, 29.94 and 24.04 folds at month 6, 25.58 and 21.41 folds at month 12, respectively (*p* < 0.0001) (Figure 2 and Table S2). No significant difference of the NAb GMTs against live wild-type SARS-CoV-2 was found between the low-dose and high-dose aerosolised Ad5-nCoV groups at either month 3 (*p* = 0.4206), month 6 (*p* = 0.3112) or month 12 (*p* = 0.4305). However, compared with the peaking levels of NAb GMTs on day 28 after the booster dose, the NAb GMTs against live wild-type SARS-CoV-2 decreased by 72.26% and 72.01% at month 3, 83.62% and 84.64% at month 6, 89.30% and 89.52% in month 12 in the low-dose and high-dose aerosolised Ad5-nCoV groups, respectively. Similarly, in the CoronaVac group, the NAb GMTs decreased by 70.78% at month 3, 85.04% month 6, and 88.54% at month 12.

The low-dose and high-dose aerosolised Ad5-nCoV groups had substantially higher GMFIs from the baseline Nab GMTs before the boosting than did the CoronaVac group at month 3 (139.58 [95% CI 100.75, 193.40] and 110.18 [95% CI 76.73, 158.23] vs. 5.56 [95% CI 3.63, 8.51]), month 6 (84.14 [95% CI 58.17, 121.70], 60.41 [95% CI 39.95, 91.33] vs. 2.80 [95% CI 1.96, 4.00]) and month 12 (47.67 [95% CI 33.44, 67.96], 39.83 [95% CI 26.33, 60.26] vs. 1.80 [95% CI 1.02, 3.16]) (Table S2). In addition, all participants (100%) in the low-dose and high-dose groups were serum seropositive for NAb GMTs against live wild-type SARS-CoV-2 at all pre-specified time points till month 12, while 85.00% (34/40), 66.67% (26/39) and 61.54% (8/13) of the participants in the CoronaVac group had seropositivity at month 3, month 6 and month 12, respectively (Table S2).

### NAbs against live omicron BA.1 subvariant

The NAb GMTs against live omicron BA.1 of the low-dose and the high-dose aerosolised Ad5-nCoV groups were 51.98 (95% CI 37.23, 72.58) and 23.07 (95% CI 15.68, 33.95) at day 28, 27.86 (95% CI 18.81, 41.26) and 23.27 (95% CI 16.24, 33.35) at month 3, 16.00 (95% CI 10.88, 23.53) and 11.99 (95% CI 8.55, 16.81) at month 6, respectively. In the low-dose aerosolised Ad5-nCoV group, the NAb GMT against live omicron BA.1 declined by 46.60% and 69.22% at 3 and 6 months, respectively, compared with the peaking levels at day 28. While, in the high-dose aerosolised Ad5-nCoV group, the NAb GMT increased by 0.87% (23.27 [95% CI 16.24, 33.35]) at month 3 and decreased by 48.03% (11.99 [95% CI 8.55, 16.81]) at month 6, compared with those at day 28 (Figure 2). In contrast, in the CoronaVac group, nearly all participants showed no detectable NAbs against omicron BA.1 at any time point (Figure 2 and Table S3). At day 28, the low-dose aerosolised Ad5-nCoV group demonstrated a higher NAbs against omicron BA.1 than did the high-dose group (*p* = 0.0018). However, no significant difference between the two aerosolised Ad5-nCoV groups at either month 3 (*p* = 0.4983) or month 6 (*p* = 0.2593). In terms of the seropositivity for NAbs against omicron BA.1, no significant differences were noted between the two aerosolised Ad5-nCoV groups at either day 28 (*p* = 0.7015), month 3 (*p* = 0.7385), or month 6 (*p* = 0.8874).

### NAbs against omicron BA.4/5 subvariant pseudovirus

**Figure 3.**
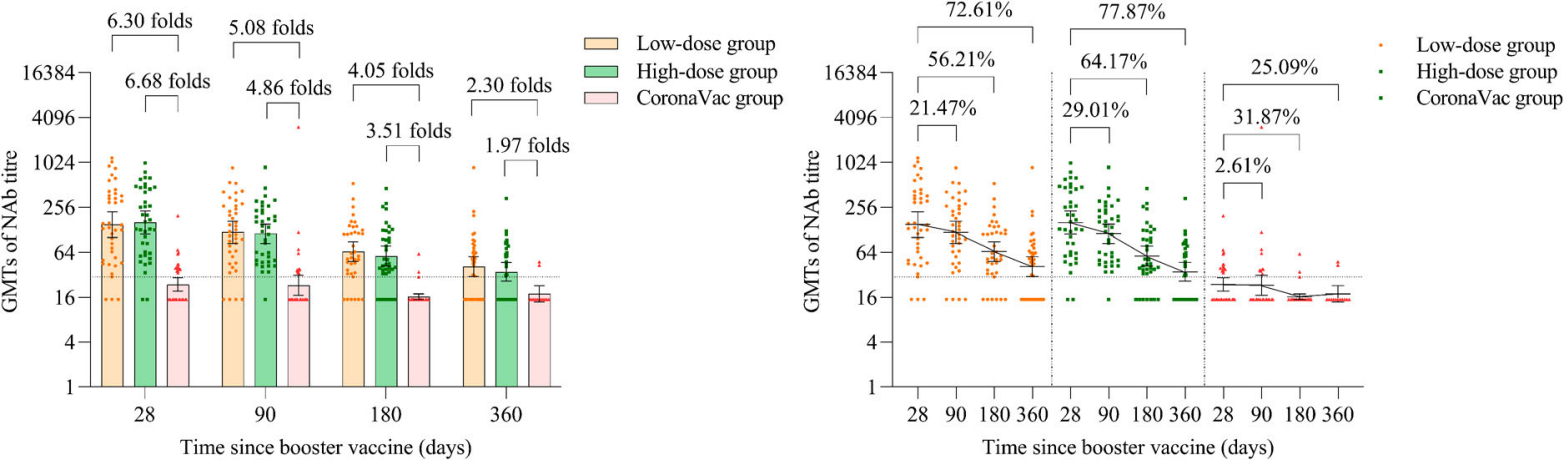
Neutralizing antibodies against omicron BA.4/5 pseudovirus after a booster vaccination. Horizontal bars show GMTs and error bars show 95% confidence intervals. Data (folds) above the bars are the NAb GMT ratios of the homologous boost group to the heterologous boost group. Long lines connecting the GMTs of adjacent groups indicate trends in neutralizing antibodies over days after a booster vaccination. Data (%) above the bars show the percent reduction in GMTs of NAb titres at day 90, day 180 and day 360 post-boost compared to day 28. All horizontal dotted lines denote the cutoff levels for positivity (1:30). Data below the cutoff level were assigned half the limit. GMT = geometric mean titre in serum. NAb = neutralizing antibody.

The GMTs of NAb against omicron BA.4/5 pseudovirus in the low-dose and high-dose aerosolised Ad5-nCoV groups were 149.58 (95% CI 101.03, 221.45) and 158.52 (95% CI 111.36, 225.66) at day 28, 117.47 (95% CI 83.65, 164.96) and 112.53 (95% CI 83.77, 151.16) at month 3, 65.50 (95% CI 48.31, 88.81) and 56.80 (95% CI 41.65, 77.46) at month 6, 40.97 (95% CI 30.15, 55.67) and 35.08 (95% CI 26.31, 46.77) at month 12, respectively. Compared with 28 days, in the low-dose and high-dose aerosolised Ad5-nCoV groups, the GMTs of NAb against omicron BA.4/5 pseudovirus decayed by 21.47% and 29.01% at month 3, 56.21% and 64.17% at month 6, 72.61% and 77.87% at month 12, respectively. In the CoronaVac group, GMTs of NAb against omicron BA.4/5 pseudovirus were below the lower limit of detection at all the time points (Table 1 and Figure 3). Significantly higher seropositivity of the participants was noted in the low-dose and high-dose aerosolised Ad5-nCoV groups as compared with that in the CoronaVac group from month 3 to month 12 (Table 1).

**Table 1.** GMT, seropositivity rate, and GMT ratio of neutralizing antibodies to omicron BA.4/5 subvariant pseudovirus after a booster vaccination.

	Low-dose group (*n* = 40)	*p* Value*	High-dose group (*n* = 40)	*p* Value**	CoronaVac group (*n* = 40)	*p* Value***
Day 28						
GMT	149.58 (101.03, 221.45)	<0.0001	158.52 (111.36, 225.66)	<0.0001	23.75 (19.41, 29.05)	0.8247
Seropositivity	92.50 (79.61, 98.43)	<0.0001	94.87 (82.68, 99.37)	<0.0001	40.00 (24.86, 56.67)	1.0000
GMT ratio	6.30 (4.60, 9.98)	–	6.68 (4.87, 10.60)	–	ref	–
Month 3						
GMT	117.47 (83.65, 164.96)	<0.0001	112.53 (83.77, 151.16)	<0.0001	23.13 (17.05, 31.38)	0.8478
Seropositivity	90.00 (76.34, 97.21)	<0.0001	97.37 (86.19, 99.93)	<0.0001	30.00 (16.56, 46.53)	0.1842
GMT ratio	5.08 (3.72, 8.00)	–	4.86 (3.57, 7.64)	–	ref	–
Month 6						
GMT	65.50 (48.31, 88.81)	<0.0001	56.80 (41.65, 77.46)	<0.0001	16.18 (14.78, 17.70)	0.5084
Seropositivity	84.21 (68.75, 93.98)	<0.0001	81.08 (64.84, 92.04)	<0.0001	7.69 (1.62, 20.87)	0.7204
GMT ratio	4.05 (2.84, 7.01)	–	3.51 (2.49, 5.97)	–	ref	–
Month 12						
GMT	40.97 (30.15, 55.67)	0.0038	35.08 (26.31, 46.77)	0.0100	17.79 (13.83, 22.88)	0.4582
Seropositivity	62.50 (45.80, 77.27)	0.0032	55.26 (38.30, 71.38)	0.0126	15.38 (1.92, 45.45)	0.5160
GMT ratio	2.30 (1.71, 3.54)	–	1.97 (1.49, 2.93)	–	ref	–

Notes: Data are GMT (95% CI), seropositivity (%, 95% CI), GMT ratio (95% CI). GMT = geometric mean titre. Seropositivity (%): The proportion of participants whose antibody titres was defined as a detectable neutralizing antibody titre ≥1:30. Data below the cutoff level were assigned half the limit. *The *p* values of this column are the results of comparison between low-dose aerosolised vaccine group and inactivated vaccine group. **The *p* values of this column are the results of comparison between high-dose aerosolised vaccine group and inactivated vaccine group. ***The *p* values of this column are the results of comparison between low-dose aerosolised vaccine group and high-dose aerosolised vaccine group. GMT ratio = heterologous boost group/homologous boost group. Measurements on day 28 were taken 28 days after the booster vaccination.

### Wild-type SARS-CoV-2 RBD-specific IgG antibodies

**Figure 4.**
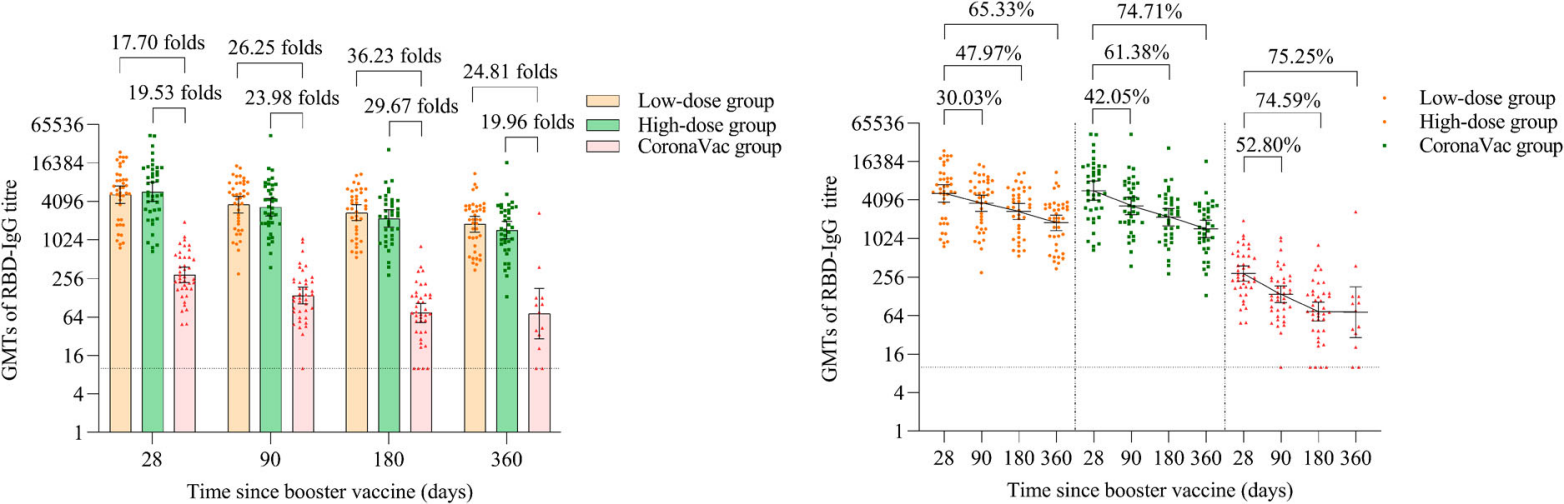
Wild-type SARS-CoV-2 RBD-specific IgG antibodies after a booster vaccination. Horizontal bars show GMTs and error bars show 95% confidence intervals. Data (folds) above the bars are the NAb GMT ratios of the homologous boost group to the heterologous boost group. Long lines connecting the GMTs of adjacent groups indicate trends in RBD-IgG antibodies over days after a booster vaccination. Data (%) above the bars show the percent reduction in GMTs of RBD-IgG antibody titres at day 90, day 180 and day 360 post-boost compared to day 28. All horizontal dotted lines denote the detectable cutoff titres of 1:10. GMT = geometric mean titre in serum. RBD-IgG = receptor-binding domain (RBD)-specific IgG antibodies.

The GMTs of wild-type SARS-CoV-2 RBD-specific IgG antibodies in the low-dose and high-dose aerosolised Ad5-nCoV groups were 5210.84 (95% CI 3797.85, 7149.54), 5743.39 (95% CI 4059.90, 8124.94) at day 28, 3646.17 (95% CI 2706.95, 4911.28), 3328.55 (95% CI 2429.74, 4559.84) at month 3, 2711.25 (95% CI 2040.90, 3601.79), 2218.17 (95% CI 1621.50, 3034.39) at month 6, 1806.39 (95% CI 1363.49, 2393.14), 1452.29 (95% CI 1062.17, 1985.69) at month 12. Compared with the peaking RBD-specific IgG at day 28 after the booster dose, the GMTs of RBD-specific IgG antibodies in the low-dose and high-dose aerosolised Ad5-nCoV groups decreased by 30.30% and 42.05% at month 3, 47.97% and 61.38% at month 6, 65.33% and 74.71% at month 12, respectively. Likewise, in the CoronaVac group, the NAb GMTs decreased by 52.80%, 74.59% and 75.25%, respectively, at the same time points. However, the GMTs of the low-dose and high-dose aerosolised Ad5-nCoV groups were 26.25 and 23.98 folds higher at month 3, 36.23 and 29.67 folds higher at month 6, 24.81 and 19.96 folds higher at month 12, as compared with those of the CoronaVac group respectively (*p* < 0.0001) (Figure 4 and Table S4). There was no significant difference in RBD-specific IgG between the two aerosolised Ad5-nCoV groups at either month 3 (*p* = 0.6712), month 6 (*p* = 0.3386) or month 12 (*p* = 0.2960).

GMFIs of the low-dose and high-dose aerosolised Ad5-nCoV groups were significantly higher compared with that of the CoronaVac group throughout the time points till month 12 (Table S4).

### SAEs and pregnancy events

A total of four (0.95%) participants reported SAEs from day 28 till month 12. All of the participants with SAEs were resolved within 5 days after the hospitalization. All the four SAEs were determined to be unrelated to vaccination (Table S5). In addition, five female participants became pregnant during the follow-up period. No adverse pregnancy outcomes or fetal abnormalities were observed during the 12-month follow-up after the booster vaccination (Table S6).

## Discussion

The aerosolized Ad5-nCoV is the first aerosolized COVID-19 vaccine administrated by oral inhalation authorized for emergency use as a booster dose following the primary series of inactivated COVID-19 vaccines. The initial study reported previously indicated that the heterologous aerosolized Ad5-nCoV plus two-dose CoronaVac was more immunogenic than three-dose CoronaVac within 28 days post-vaccination in Chinese adults aged 18 years and older [11]. In this report, we reported the humoral immune responses in terms of the NAb GMTs against the wild-type SARS-CoV-2, the omicron BA.4/5 and the omicron BA.1 subvariant at day 28, month 6 and/or month 12 as the final report of the trial. Our results showed that after receiving the heterologous boost with 0.1 or 0.2 mL aerosolized Ad5-CoV, participants had persistently higher NAb GMTs to both the prototype and omicron variants than the peak NAb levels achieved by day 28 after receiving the homologous boost with CoronaVac. Moreover, we found no vaccine-related long-term safety concerns associated with aerosolized Ad5-CoV. This is the first study to show the antibody persistence and the safety data after heterologous boosting with orally aerosolised Ad5-nCoV in individuals primed with two-dose CoronaVac previously.

Although the quick waning of NAbs along with time has been found after the primary or boosting immunization with all kinds of COVID-19 vaccines, the decay rates of neutralizing response associated with different COVID-19 vaccines could be varied [13,14]. However, in this study, we found that the decay patterns of the NAb responses after the peaking following the boosting with aerosolized Ad5-nCoV within 12 months were similar to that following the CoronaVac boosting. Furthermore, this decline of NAb responses was obvious within the first 6 months, and much slower after that, which is consistent with that observed in individuals after receiving other vectored based or virus-like particle COVID-19 vaccines [15].

Since considerable escape of the omicron variants to NAb induced by the first generation of COVID-19 vaccine against the prototype strain has been wildly reported [16], it is necessary to analyse the NAb responses to both the omicron BA.1 and BA.4/5. Our results showed that participants receiving a booster dose of low-dose or high-dose aerosolized Ad5-nCoV had the seropositivity of 92.5% for NAbs against omicron BA.1 at day 28, then decreased to 72.2% at month 6, and 97.4% for NAbs against omicron BA.4/5 at day 28, then decreased to 55.3% at month 12. While nearly all participants had no detective NAbs against omicron variants at any time points before or after the homologous boost with CoronaVac.

In this study, our data firstly demonstrated the NAb responses against omicron variants persisted for at least 12 months produced by orally administered aerosolised Ad5-nCoV following two-dose CoronaVac priming. NAbs have been proven to be highly predictive of SARS-CoV-2 immune protection [17], and vaccine efficacy increased as NAb levels increased [18]. Consequently, our study suggested that although the omicron variant evaded NAb responses elicited by the homologous CoronaVac boost regimen, the heterologous boost regimen may provide a broad spectrum of antibodies against both omicron BA.1 and BA.4/5 subvariants, which could be beneficial to fight against other predominant SARS-CoV-2 variants in the future and to slow the immune escape of SARS-CoV-2 variants in the face of the selective pressure [19].

Previously, an increased risk of the rare and potentially fatal thrombosis with thrombocytopenia syndrome has been found associated with recipients of the adenovirus viral vector vaccine (ChAdOx1 nCoV-19) [20,21]. Other studies on another adenovirus viral vector vaccine for COVID-19 (Sputnik V) have revealed a rare incidence of 0.1 cases per million doses associated with the vaccination of Ad5-vectored COVID-19 vaccine [22]. However, we found no thrombosis or other vaccine-related SAEs in this study. Since the sample size of this study is likely to be too small to identify any rare adverse reactions following the immunization, further surveillance of adverse reactions associated with the aerosolised Ad5-nCoV is needed in a large population.

This study had several limitations. Firstly, mucosal immune responses were not provided because no saliva or mucosal immunity samples were collected per protocol. Secondly, vaccine efficacy or vaccine effectiveness against SARS-CoV-2 was not presented because of no widespread COVID-19 outbreak during the study period. Additionally, the influencing factors of antibody persistence had not been analysed due to the small sample size. However, previous studies found that prior infection and age influence the magnitude and persistence of antibody responses [23–25]. Finally, only a small proportion of participants aged 60 years and older were involved in this trial. The antibody persistence of heterologous aerosolized Ad5-nCoV regimens in 80 years of age or older and other special populations will be required.

Recently, low-dose aerosolized Ad5-nCoV at 0.1 mL has been authorized for emergency use as a booster in China and has initiated the deployment of massive vaccination campaign. Each aerosolized Ad5-nCoV vaccination uses only one-fifth intramuscular injection dose of the originally licensed Ad5-nCoV, which is significantly beneficial for addressing the challenge that limited resource in vaccine distribution in many parts of the world needs to be addressed to improve global access to the COVID-19 vaccines. In addition, compared to the intramuscular COVID-19 vaccines, the aerosolized Ad5-nCoV is supposed to be able to elicit respiratory mucosal immunity, which could potentially provide additional protection by eliciting innate and adaptive immunity in the mucosal tissue [26].

In conclusion, our study demonstrated that heterologous orally aerosolised Ad5-nCoV as a booster following two-dose CoronaVac priming had a good long-term safety profile and was persistently more immunogenic than three-dose CoronaVac within 12 months after boosting, providing additional support for the massive COVID-19 vaccine boosting campaign.

## Supplementary Material

Supplemental Material
