# Supplementary material for: Antibody persistence and safety after heterologous boosting with orally aerosolised Ad5-nCoV in individuals primed with two-dose CoronaVac previously: 12-month analyses of a randomized controlled trial
Source: Emerg Microbes Infect. 2022 Dec 15;12(1):2155251. doi: 10.1080/22221751.2022.2155251 (PMC10519268; doi:10.1080/22221751.2022.2155251)
Supplement: Supplemental Material [file TEMI_A_2155251_SM7733.docx]

**Supplemental Online Content**

[**Table S1. Demographic characteristics of the participants in this study 2**](#_Toc112628231)

[**Table S2. Neutralizing antibodies to live wild-type SARS-CoV-2 after a booster vaccination. 3**](#_Toc112628232)

[**Table S3. Neutralizing antibodies to live omicron BA.1 subvariant after a booster vaccination. 5**](#_Toc112628233)

[**Table S4. Wild-type SARS-CoV-2 RBD-specific IgG antibodies after a booster vaccination. 6**](#_Toc112628234)

[**Table S5. Characteristics of serious adverse events during the study 7**](#_Toc112628235)

[**Table S6. Characteristics of pregnancy events during the study 8**](#_Toc112628236)

# Table S1. Demographic characteristics of the participants in this study

|  | **Immunogenicity analysis cohort** | | |  | **Safety analysis cohort** | | |
| --- | --- | --- | --- | --- | --- | --- | --- |
|  | Low-dose group (n=40) | High-dose group (n=40) | CoronaVac group (n=40) |  | Low-dose group (n=140) | High-dose group (n=139) | CoronaVac group (n=140) |
| Gender |  |  |  |  |  |  |  |
| Male, n (%) | 17(42.50) | 20(50.00) | 15(37.50) |  | 59(42.14) | 60(43.17) | 60(42.86) |
| Female, n (%) | 23(57.50) | 20(50.00) | 25(62.50) |  | 81(57.86) | 79(56.83) | 80(57.14) |
| Age, years |  |  |  |  |  |  |  |
| 18-59, n (%) | 38(95.00) | 39(97.50) | 38(95.00) |  | 137(97.86) | 137(98.56) | 138(98.57) |
| ≥60, n (%) | 2(5.00) | 1(2.50) | 2(5.00) |  | 3(2.14) | 2(1.44) | 2(1.43) |
| mean (SD) | 38.13(11.95) | 39.83(10.04) | 40.13(11.52) |  | 40.11(10.10) | 41.04(9.74) | 41.21(9.95) |
| Months since the last prime dose of CoronaVac, median (IQR) | 5.00(4.00, 5.00) | 5.00(5.00, 5.0) | 5.00(5.00, 5.00) |  | 5.00(5.00, 5.0) | 5.00(5.00, 5.00) | 5.00(5.00, 5.00) |

Notes: SD= standard deviation. IQR=Interquartile range.

# Table S2. Neutralizing antibodies to live wild-type SARS-CoV-2 after a booster vaccination.

|  | **Low-dose group (n=40)** | ***P* value ^*^** | **High-dose group**  **(n=40)** | ***P* value ^**^** | **CoronaVac group**  **(n=40)** | ***P* value ^***^** |
| --- | --- | --- | --- | --- | --- | --- |
| Day 28 | | | | | |  |
| GMT | 1910.54(1391.98, 2622.28) | <0.0001 | 1635.03(1112.16, 2403.73) | <0.0001 | 69.79(46.25, 105.32) | 0.5265 |
| Seropositivity | 100.00(91.19, 100.00) | 1.0000 | 100.0(90.5, 100.0) | 1.0000 | 97.50(86.84, 99.94) | — |
| GMFI | 503.12(327.18, 773.67) | <0.0001 | 393.73(249.35, 621.70) | <0.0001 | 19.03(12.26, 29.54) | 0.4309 |
| GMT ratio | 27.40(22.27, 35.59) | — | 23.42(19.05, 30.40) | — | ref | — |
| Month 3 | | | | | |  |
| GMT | 530.06(412.49, 681.12) | <0.0001 | 457.57(349.40, 599.22) | <0.0001 | 20.39(14.27, 29.14) | 0.4206 |
| Seropositivity | 100.00(91.19, 100.00) | 0.0255 | 100.00(90.51, 100.01) | 0.0260 | 85.00(70.16, 94.29) | — |
| GMFI | 139.58(100.75, 193.40) | <0.0001 | 110.18(76.73, 158.23) | <0.0001 | 5.56(3.63, 8.51) | 0.3273 |
| GMT ratio | 25.97(18.25, 45.25) | — | 22.42(15.75, 38.91) | — | ref | — |
| Month 6 | | | | | |  |
| GMT | 312.88(237.71, 411.82) | <0.0001 | 251.12(178.16, 353.96) | <0.0001 | 10.44(7.78, 14.02) | 0.3112 |
| Seropositivity | 100.00(90.75, 100.00) | <0.0001 | 100.00(90.26 100.00) | <0.0001 | 66.67(49.78, 80.91) | — |
| GMFI | 84.14(58.17, 121.70) | <0.0001 | 60.41(39.95, 91.33) | <0.0001 | 2.80(1.96, 4.00) | 0.2281 |
| GMT ratio | 29.94(18.76, 74.07) | — | 24.04(15.08, 59.17) | — | ref | — |
| Month 12 |  |  |  |  |  |  |
| GMT | 204.36(152.91, 273.14) | <0.0001 | 171.38(121.27, 242.19) | <0.0001 | 8.00(4.22, 15.17) | 0.4305 |
| Seropositivity | 100.00(91.19, 100.00) | 0.0004 | 100.00(90.75, 100.00) | 0.0005 | 61.54(31.58, 86.14) | — |
| GMFI | 47.67(33.44, 67.96) | <0.0001 | 39.83(26.33, 60.26) | <0.0001 | 1.80(1.02, 3.16) | 0.5052 |
| GMT ratio | 25.58(79.37, 15.22) | — | 21.41(12.77, 66.23) | — | ref | — |

Notes: Data are GMT (95% CI), seropositivity (%, 95% CI), GMT ratio (95% CI) or GMFI (95% CI). GMT=geometric mean titer. GMFI=geometric mean fold increase. Seropositivity (%): The proportion of participants whose antibody titers was defined as a detectable neutralizing antibody titer ≥ 1:8. Data below the cutoff level were assigned half the limit. **^*^** The p values of this column are the results of comparison between low-dose aerosolised vaccine group and inactivated vaccine group. **^**^** The p values of this column are the results of comparison between high-dose aerosolised vaccine group and inactivated vaccine group. **^***^** The p values of this column are the results of comparison between low-dose aerosolised vaccine group and high-dose aerosolised vaccine group. GMT ratio=heterologous boost group/homologous boost group. Measurements on day 28 were taken 28 days after the booster vaccination.

# Table S3. Neutralizing antibodies to live omicron BA.1 subvariant after a booster vaccination.

|  | **Low-dose group**  **(n=40)** | ***P* value ^*^** | **High-dose group**  **(n=40)** | ***P* value ^**^** | **CoronaVac group**  **(n=40)** | ***P* value ^***^** |
| --- | --- | --- | --- | --- | --- | --- |
| Day 28 | | | | | |  |
| GMT | 51.98(37.23, 72.58) | <0.0001 | 23.07(15.68, 33.95) | <0.0001 | 4.07(3.93, 4.22) | 0.0018 |
| Seropositivity | 92.50(79.61, 98.43) | <0.0001 | 88.89(73.94, 96.89) | <0.0001 | 2.50(0.06, 13.16) | 0.7015 |
| GMT ratio | 12.77(6.61, 188.68) | — | 5.67(3.01, 47.85) | — | ref | — |
| Month 3 | | | | | |  |
| GMT | 27.86(18.81, 41.26) | <0.0001 | 23.27(16.24, 33.35) | <0.0001 | 4.14(3.86, 4.44) | 0.4983 |
| Seropositivity | 85.00(70.16, 94.29) | <0.0001 | 89.19(74.58, 96.97) | <0.0001 | 2.50(0.06, 13.16) | 0.7385 |
| GMT ratio | 6.73(3.56, 60.61) | — | 5.62(3.00 ,44.4) | — | ref | — |
| Month 6 | | | | | |  |
| GMT | 16.00(10.88, 23.53) | <0.0001 | 11.99(8.55, 16.81) | <0.0001 | 4.07(3.93, 4.22) | 0.2593 |
| Seropositivity | 73.68(56.90, 86.60) | <0.0001 | 72.22(54.81, 85.80) | <0.0001 | 2.56(0.06, 13.48) | 0.8874 |
| GMT ratio | 3.93(2.14, 24.39) | — | 2.95(1.65, 14.01) | — | ref | — |

Notes: Data are GMT (95% CI), seropositivity (%, 95% CI), GMT ratio (95% CI). GMT=geometric mean titer. Seropositivity (%): The proportion of participants whose antibody titers was defined as a detectable neutralizing antibody titer ≥ 1:8. Data below the cutoff level were assigned half the limit. **^*^** The p values of this column are the results of comparison between low-dose aerosolised vaccine group and inactivated vaccine group. **^**^** The p values of this column are the results of comparison between high-dose aerosolised vaccine group and inactivated vaccine group. **^***^** The p values of this column are the results of comparison between low-dose aerosolised vaccine group and high-dose aerosolised vaccine group. GMT ratio=heterologous boost group/homologous boost group. Measurements on day 28 were taken 28 days after the booster vaccination.

# Table S4. Wild-type SARS-CoV-2 RBD-specific IgG antibodies after a booster vaccination.

|  | **Low-dose group (n=40)** | ***P* value ^*^** | **High-dose group**  **(n=40)** | ***P* value ^**^** | **CoronaVac group**  **(n=40)** | ***P* value ^***^** |
| --- | --- | --- | --- | --- | --- | --- |
| Day 28 | | | | | |  |
| GMT | 5210.84(3797.85, 7149.54) | <0.0001 | 5743.39(4059.90, 8124.94) | <0.0001 | 294.16(223.66, 386.88) | 0.6762 |
| GMFI | 203.93(127.10, 327.22) | <0.0001 | 198.36(121.36, 324.20) | <0.0001 | 14.49(9.84, 21.33) | 0.9347 |
| GMT ratio | 17.70(15.95, 19.92) | — | 19.53(17.57, 21.98) | — | ref | — |
| Month 3 | | | | | |  |
| GMT | 3646.17(2706.95, 4911.28) | <0.0001 | 3328.55(2429.74, 4559.84) | <0.0001 | 138.83(102.94, 187.23) | 0.6712 |
| GMFI | 142.70(92.80, 219.43) | <0.0001 | 120.24(76.34, 189.41) | <0.0001 | 6.84(4.52, 10.34) | 0.5811 |
| GMT ratio | 26.25(22.57, 31.35) | — | 23.98(20.62, 28.65) | — | ref | — |
| Month 6 | | | | | |  |
| GMT | 2711.25(2040.90, 3601.79) | <0.0001 | 2218.17(1621.50, 3034.39) | <0.0001 | 74.74(53.04, 105.31) | 0.3386 |
| GMFI | 108.69(71.26, 165.77) | <0.0001 | 80.37(50.30, 128.40) | <0.0001 | 3.62(2.33, 5.61) | 0.3344 |
| GMT ratio | 36.23(29.67, 46.73) | — | 29.67(24.27, 38.17) | — | ref | — |
| Month 12 |  |  |  |  |  |  |
| GMT | 1806.39(1363.49, 2393.14) | <0.0001 | 1452.29(1062.17, 1985.69) | <0.0001 | 72.80(29.21, 181.39) | 0.2960 |
| GMFI | 70.69(46.77, 106.85) | <0.0001 | 52.46(33.63, 81.86) | <0.0001 | 3.70(1.72, 7.97) | 0.3225 |
| GMT ratio | 24.81(20.24, 32.05) | — | 19.96(16.29, 25.71) | — | ref | — |

Notes: Data are GMT (95% CI), GMFI (95% CI) or GMT ratio (95%CI). RBD-IgG antibodies were measured by indirect ELISA assay with a cutoff titre of 1:10. GMT=geometric mean titer. GMFI=geometric mean fold increase. GMT ratio=heterologous boost group/homologous boost group. **^*^** The p values of this column are the results of comparison between low-dose aerosol vaccine group and inactivated vaccine group. **^**^** The p values of this column are the results of comparison between high-dose aerosol vaccine group and inactivated vaccine group. **^***^** The p values of this column are the results of comparison between low-dose aerosolised vaccine group and high-dose aerosolised vaccine group. Measurements on day 28 were taken 28 days after the booster dose.

# Table S5. Characteristics of serious adverse events during the study

| **Subject ID** | **Group** | **Gender** | **Age** | **Time of onset**  **(days after the booster vaccination)** | **Reason for hospitalization** | **Underling Disease** | **Days of hospital stay** | **Treatment** | **SAE Outcome** | **Relationship with vaccines** |
| --- | --- | --- | --- | --- | --- | --- | --- | --- | --- | --- |
| V250 | Low-dose group | Male | 60 | 28 | Lung nodules for 6 years | 1. Lung nodules; 2. Hypothyroidism; 3. Hypertension | 4 | Right middle lobectomy | Improved | Irrelevant |
| V108 | High-dose group | Female | 51 | 82 | Repeated dizziness for 2 years | 1. Vertigo;  2. After ectopic pregnancy;  3. Thyroid nodules;  4. Erosive gastritis;  5. Fatty liver. | 5 | Symptom treatment such as anti dizziness, acid suppression, and protection of gastric mucosa | Improved | Irrelevant |
| V400 | Low-dose group | Female | 46 | 132 | Repeated headaches for more than 20 years | Central atrial septal defect (foramen ovale) | 4 | Percutaneous foramen ovale closure | Improved | Irrelevant |
| V405 | CoronaVac group | Male | 53 | 146 | Cough and asthma for more than 2 months | 1. Cardiac insufficiency  2. Coronary atherosclerotic heart disease 3. Bilateral pleural effusion 4. Atrial fibrillation 5. Pericardial effusion 6. Type 2 diabetes 7. Hypertension | 5 | Symptomatic treatment such as anti-infection, anticoagulation, ventricular rate control, and cardiac load reduction | Improved | Irrelevant |

# Table S6. Characteristics of pregnancy events during the study

| **Subject ID** | **Group** | **Age**  **(years)** | **Height**  **(cm)** | **Weight**  **(kg)** | **Last menstrual period (days after the booster vaccination)** | **Length of pregnancy when reporting to investigators** | **Concomitant medication** |
| --- | --- | --- | --- | --- | --- | --- | --- |
| V390 | Low-dose group | 30 | 158 | 48 | 117 | 8 weeks | None |
| V306 | Low-dose group | 32 | 160 | 50 | 38 | 21 weeks | None |
| V271 | Low-dose group | 24 | 166 | 50 | 91 | 14 weeks + 1 day | None |
| V345 | Low-dose group | 30 | 165 | 51 | 292 | 10 weeks+4 days | None |
| V085 | CoronaVac group | 31 | 172 | 60 | 33 | 21 weeks | None |
